# Data for proteomic analysis of ATP-binding proteins and kinase inhibitor target proteins using an ATP probe

**DOI:** 10.1016/j.dib.2015.10.018

**Published:** 2015-10-26

**Authors:** Jun Adachi, Marina Kishida, Shio Watanabe, Yuuki Hashimoto, Kazuna Fukamizu, Takeshi Tomonaga

**Affiliations:** Laboratory of Proteome Research, National Institute of Biomedical Innovation, Health and Nutrition, Ibaraki, Osaka, Japan

## Abstract

Interactions between ATP and ATP-binding proteins (ATPome) are common and are required for most cellular processes. Thus, it is clearly important to identify and quantify these interactions for understanding basic cellular mechanisms and the pathogenesis of various diseases. We used an ATP competition assay (competition between ATP and acyl-ATP probes) that enabled us to distinguish specific ATP-binding proteins from non-specific proteins (Adachi et al., 2014) [1]. As a result, we identified 539 proteins, including 178 novel ATP-binding protein candidates. We also established an ATPome selectivity profiling method for kinase inhibitors using our cataloged ATPome list. Normally only kinome selectivity is profiled in selectivity profiling of kinase inhibitors. In this data, we expand the profiled targets from the kinome to the ATPome through performance of ATPome selectivity profiling and obtained target profiles of staurosporine and (S)-crizotinib. The data accompanying the manuscript on this approach (Adachi et al., 2014) [1] have been deposited to the ProteomeXchange with identifier PXD001200.

**Specifications** **table**

**Table:** 

Subject area	Chemical biology
More specific subject area	ATP binding proteins and mass spectrometry(MS)
Type of data	Table
How data was acquired	MS: Data-dependent acquisition acquired on Q-Exactive (Thermo)
Data format	Analyzed
Experimental factors	SILAC labeled-Hela-S3 cells
Experimental features	ATP binding proteins (ATPome) were labeled by ATP probe, followed by tryptic digest. Labeled-peptides were enriched and analyzed by LC-MS/MS.
Data source location	National Institute of Biomedical Innovation, Health and Nutrition, Osaka, Japan
Data accessibility	Deposited to the ProteomeXchange with identifier PXD001200 (http://www.proteomexchange.org/feeditems/new-proteomexchange-dataset-pxd001200)

**Value of the data**

• Large-scale and specific ATP binding protein catalog was obtained by ATP competition assay (competition between ATP and acyl-ATP probes).
• 539 proteins, including 178 novel ATP-binding protein candidates were identified. We also found multiple ATP-competitive sites for several kinases, including epidermal growth factor receptor.
• As described in detail in the accompanying manuscript (DOI: 10.1021/pr500845u), we proved the concept to expanding the profiled targets from the kinome to the ATPome through performance of ATPome selectivity profiling.

## 1 Data

In the present work, we provide the ATP binding protein catalog generated by ATP competition assay including kinases and other ATP binding proteins [1]. We include a table containing quantitative and qualitative information about ATP binding proteins and a supplemental figure in which all the identified kinases are mapped.

## 2 Materials and methods

### 2.1 Cell cultures

Hela-S3 cells were grown in DMEM with 10% fetal bovine serum plus antibiotics in 10% CO_2_ at 37 °C. For SILAC labeling, Hela-S3 cells were cultured in DMEM supplemented with 10% dialyzed fetal bovine serum and either 28.0 mg/L normal isotopic abundance arginine and 48.7 mg/L normal isotopic abundance lysine (Light) or 28.0 mg/L arginine with six ^13^C and four ^15^N atoms and 48.7 mg/L lysine with six ^13^C and two ^15^N atoms (Heavy) [2]. Labeling efficiency was confirmed after five passages.

### 2.2 Sample preparation

Approximately 5×10^8^ Hela-S3 cells after each of the light and heavy SILAC labeling conditions were harvested with 5 ml of ice-cold lysis buffer (25 mM Tris pH 7.5, 150 mM NaCl, 1% CHAPS, 1% Nonidet P-40, protease inhibitor cocktail and phosphatase inhibitor cocktail). Cell lysate was incubated on ice for 15 min, followed by centrifugation at 16000*g* at 4 °C for 5 min. Free endogenous ATP, ADP, and small molecules in supernatants were removed by gel filtration using a Zeba spin column (Pierce). Halt protease/phosphatase inhibitor cocktail was then added to the sample. Cell lysate was diluted with reaction buffer (20 mM Hepes pH 7.5, 150 mM NaCl, 0.1% Triton X100) to a final protein concentration of 4 mg/mL. MnCl_2_ was added to 250 μL of lysates at a final concentration of 20 mM. The labeling reaction in the ATP-competition assay was carried out at a final concentration of 5 μM ATP probe at room temperature with gentle shaking for 10 min, following preincubation with ATP for 10 min. After the reaction, the sample was denatured by 5 M urea, reduced with DTT (5 mM final concentration), and alkylated with iodoacetamide (20 mM final concentration). The labeling reaction in the kinase inhibitor experiment was performed similarly, with minor modifications. Kinase inhibitors were preincubated for 10 min with SILAC-labeled “Light” lysate (1 mg protein), while SILAC-labeled “Heavy” lysate (1 mg protein) were not treated with kinase inhibitors. After denaturation with urea, “Heavy” lysate was spiked into an equal amount of “Light” lysate. After the alkylation step, the solution was substituted by digestion buffer by gel filtration followed by digestion at 37 °C overnight with sequencing-grade trypsin at an enzyme/substrate ratio of 1:100. Labeled peptides were enriched by immobilized streptavidin agarose resin. Prior to enrichment, 50 μL of slurry was washed 3 times with 500 μL of elution buffer (50% acetonitrile, 0.1% TFA) and 3 times with 500 μL of digestion buffer. After addition to the digested peptide solution, the mixture was incubated at room temperature for 1 h with gentle rotation. To remove the unbound peptides, agarose resin was extensively washed with 2 ml of washing buffer (25 mM Tris–HCl pH 7.4, 150 mM NaCl, 1 mM EDTA, 1% Nonidet P-40 and 5% glycerol), 2 ml of PBS, and 2 ml of pure H_2_O. After washing, labeled peptides were eluted with 150 μL of elution buffer (50% ACN, 0.1% TFA) twice and dried in a Speed-vac. Peptides were dissolved in 50 μL of 2 M urea and 1% TFA and desalted using Stage Tips [3]. Each experiment was performed in triplicate or quadruplicate.

### 2.3 LC–MS/MS analysis

All nanoflow LC–MS/MS experiments were performed on a Q Exactive mass spectrometer (Thermo Scientific). Data were acquired in data-dependent mode using Xcalibur software. The precursor ion scan MS spectra (*m*/*z* 350–1800) were acquired in the Orbitrap with 70,000 resolution after accumulation of ions to a 3×10^6^ target value. The twelve most intensive ions were isolated and fragmented in the octopole collision cell by higher-energy collisional dissociation (HCD) with a maximum injection time of 120 ms and 35,000 resolution. In data-dependent LC/MS^2^ experiments dynamic exclusion was used with 20-s exclusion duration. Typical mass spectrometric conditions were as follows: spray voltage, 2 kV; no sheath and auxiliary gas flow; heated capillary temperature, 250 °C; normalized HCD collision energy 25%. The MS/MS ion selection threshold was set to 5×10^4^ counts. A 3.0 Da isolation width was chosen.

### 2.4 Data analysis

All raw files were processed by MaxQuant software suite (version 1.3.0.5) supported by the Andromeda search engine for peptide identification [4]. MaxQuant was used to score peptides for identification based on a search with an initial allowed mass deviation of the precursor ion of up to 7 ppm. The allowed fragment mass deviation was 20 ppm. Peak lists were searched against a concatenated forward and reversed version of the the UniProt human database (release 2011_11) combined with 262 common contaminants was performed using the Andromeda search engine. Enzyme specificity was set as C-terminal to Arg and Lys with allowed cleavage at proline bonds and a maximum of three missed cleavages. Carbamidomethylation of cysteine was set as a fixed modification and methionine oxidation and desthiobiotinylated lysine (+196.1212 Da) as variable modifications. Peptides and proteins were accepted with a false discovery rate of <1%. Feature matching between raw files was enabled for ATP competition datasets using a retention time window of 2 min. We removed peptides that were inhibited by <80% at 10 μM ATP in order to extract ATP-competitive peptides/sites from label-free quantitation data. Peptides with quantitation values that were not reproducible were also rejected, based on an average of <0.5 for three correlation coefficients of intensity values in triplicate experiments (correlation coefficients of experiments 1 and 2, 2 and 3, 3 and 1); 89.6% of peptides were rejected by this filter. MS spectra for all ATP-competitive peptides were checked for missed assignments by matching between raw files, and 946 peptides (4.3% of all peptides) passed all criteria and were recognized as ATP-competitive peptides. In the kinase inhibitor experiment, spike-in SILAC quantitation was used instead of label-free quantitation. Peptides inhibited by >50% at 10 μM staurosporine were accepted. MS spectra for all target peptides were checked manually.
