# Supplementary figures and images for: Data for proteomic analysis of ATP-binding proteins and kinase inhibitor target proteins using an ATP probe
Source: Data Brief. 2015 Oct 26;5:726–9. doi: 10.1016/j.dib.2015.10.018 (PMC4659801; doi:10.1016/j.dib.2015.10.018)

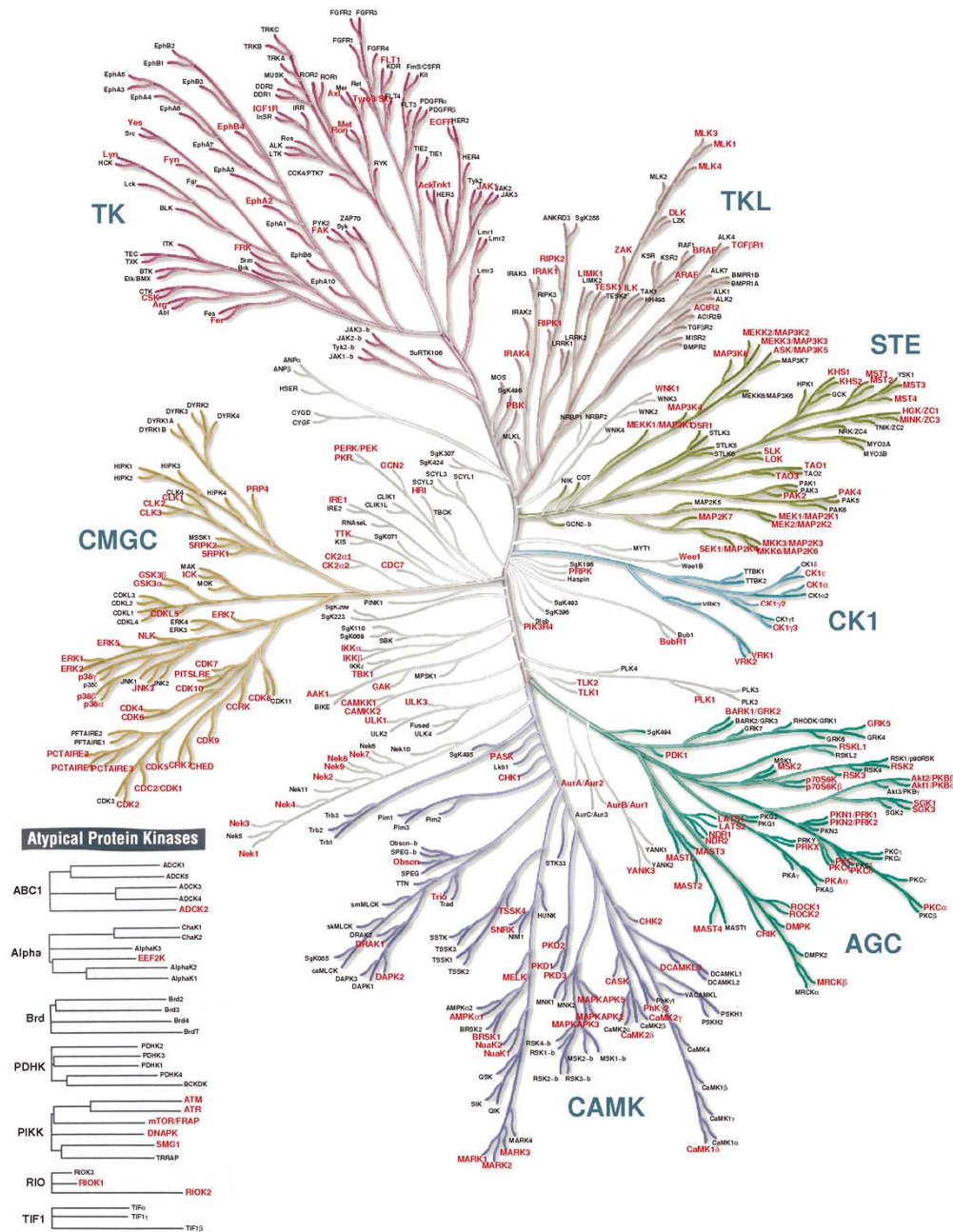

Supplementary Figure1 Identified Kinases in this study are shown in red.

Supplement: Supplementary file 2 — Supplementary material [file mmc2.pdf]
